# Supplementary material for: Inflammatory factors and risk of lung adenocarcinoma: a Mendelian randomization study mediated by blood metabolites
Source: Front Endocrinol (Lausanne). 2024 Aug 27;15:1446863. doi: 10.3389/fendo.2024.1446863 (PMC11384989; doi:10.3389/fendo.2024.1446863)
Supplement: Supplementary file 1 [file DataSheet1.zip › supplementary files/S2/GCST90200957/sensitivity-analysis.pdf]

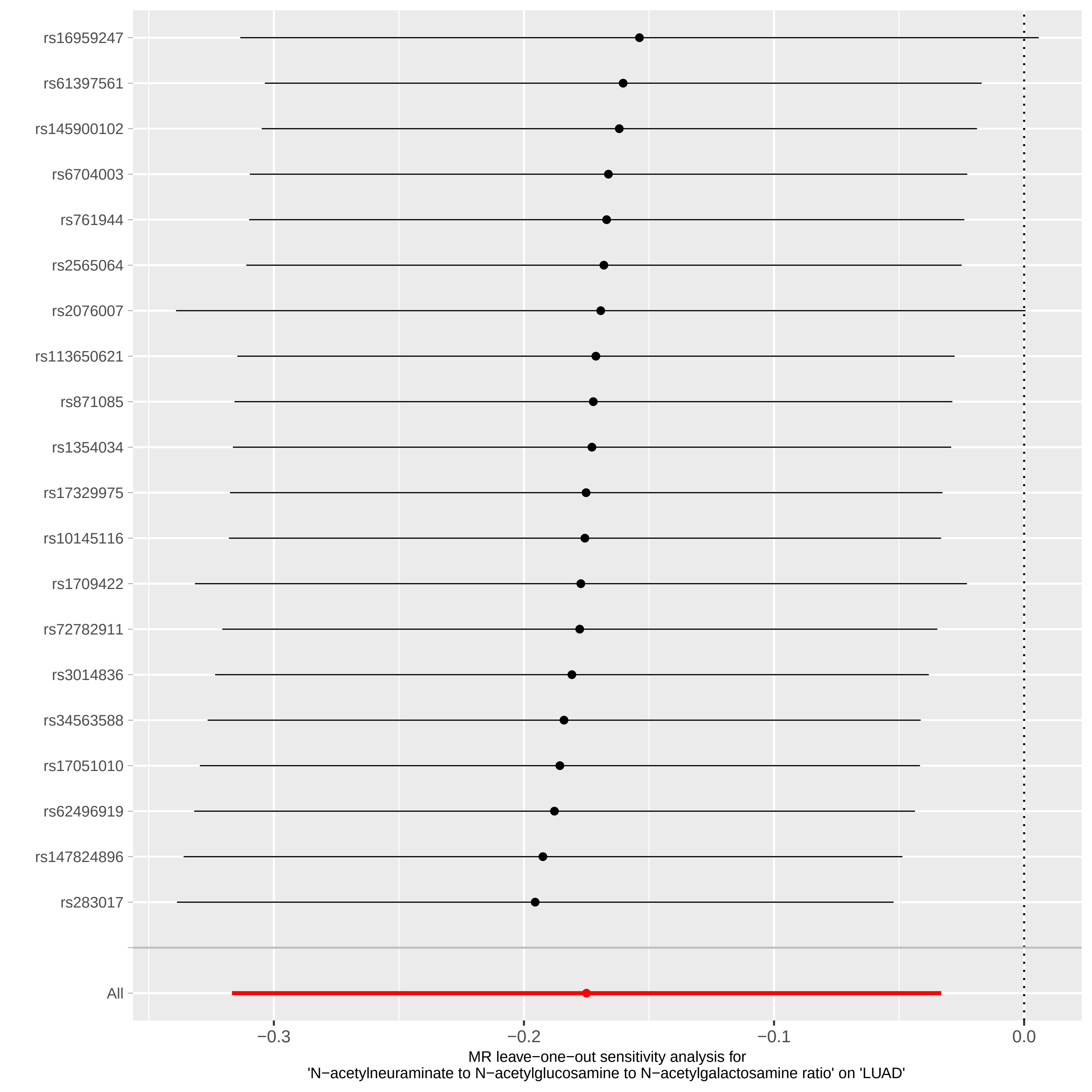
rs16959247
rs61397561
rs145900102
rs6704003
rs761944
rs2565064
rs2076007
rs113650621
rs871085
rs1354034
rs17329975
rs10145116
rs1709422
rs72782911
rs3014836
rs34563588
rs17051010
rs62496919
rs147824896
rs283017
All
−0.3
−0.2
−0.1
0.0
MR leave−one−out sensitivity analysis for
'N−acetylneuraminate to N−acetylglucosamine to N−acetylgalactosamine ratio' on 'LUAD'
